# View Normalization of Object Size in the Right Parietal Cortex

**DOI:** 10.3390/vision6030041

**Published:** 2022-07-01

**Authors:** Sylvia Hoba, Gereon R. Fink, Hang Zeng, Ralph Weidner

**Affiliations:** 1 Institute of Neuroscience and Medicine, INM-3, Research Center Jülich, 52425 Jülich, Germany; sylviahoba@mailbox.org (S.H.); g.r.fink@fz-juelich.de (G.R.F.); 2 Department of Neurology, University Hospital Cologne, Cologne University, 50937 Cologne, Germany; 3 Center for Educational Science and Technology, Beijing Normal University at Zhuhai, Zhuhai 519087, China

**Keywords:** visual perception, familiar size, right IPS, fMRI

## Abstract

Prior knowledge alters perception already on early levels of processing. For instance, judging the display size of an object is affected by its familiar size. Using functional magnetic resonance imaging, we investigated the neural processes involved in resolving ambiguities between familiar object size and physical object size in 33 healthy human subjects. The familiar size was either small or large, and the object was displayed as either small or large. Thus, the size of the displayed object was either congruent or incongruent with its internally stored canonical size representation. Subjects were asked to indicate where the stimuli appeared on the screen as quickly and accurately as possible, thereby ensuring that differential activations cannot be ascribed to explicit object size judgments. Incongruent (relative to congruent) object displays were associated with enhanced activation of the right intraparietal sulcus (IPS). These data are consistent with but extend previous patient studies, which found the right parietal cortex involved in matching visual objects presented atypically to prototypical object representations, suggesting that the right IPS supports view normalization of objects. In a second experiment, using a parametric design, a region-of-interest analysis supported this notion by showing that increases in size mismatch between the displayed size of an object and its familiar viewing size were associated with an increased right IPS activation. We conclude that the right IPS performs view normalization of mismatched information about the internally stored prototypical size and the current viewing size of an object.

## 1. Introduction

The human visual system handles a large amount of information by applying highly efficient processing strategies. For instance, computationally expensive calculations are required to identify objects within a cluttered background, to recognize their identity, and to infer additional information such as location or size. These computations are accomplished by combining different sources of information: incoming bottom-up information, such as shape or texture, and top-down information such as learned image dependencies are combined to derive likely interpretations or likely object features [1].

For instance, objects of a specific category are typically seen at specific distances. Hence, perceiving an object from a specific category typically involves several optical low features, such as the size of its representation on the retina, which depends on both the object’s physical size and its viewing distance. Once internalized, these statistics are used to resolve ambiguities inherent in a retinal image [2,3,4]. The effect of learned image statistics is illustrated by the concept of canonical size. Canonical size describes that “*real-world objects have a consistent visual size at which they are drawn, imagined and preferentially viewed*” [5]. This property takes into account the context or reference frame in which a particular object is seen. An object’s canonical visual size is viewer-centered and closely linked to experience, i.e., the typical viewing distance and knowledge about its typical size. Despite being a top-down cue, familiar size is available fast enough to affect early levels of visual processing [6]. Consistently, familiar size gives rise to interference with physical size judgments: during a two-alternative forced-choice (2AFC) size-judgment task, subjects were faster to indicate the relative display size of an object when it was congruent with the real-world size (i.e., a car displayed larger than an apple, rather than vice versa) [7]. Furthermore, when depth information was restricted, objects depicted in sizes closer to their real-world size were detected significantly faster than those that were more different from their familiar size [8]. These findings illustrate that retinal and familiar object sizes interfere at common processing levels.

There are different possibilities of how these size interferences are resolved at the neural level. One possibility is that size interference involves brain regions known to code objects’ real-world size. Incongruent display-size information may particularly modulate ventral visual regions [9]. Modulations of these regions, which have been previously found to encode real-world sizes of familiar objects, would suggest that size mismatches are resolved during the encoding stage of object representations.

Alternatively, incongruent size information may emerge at a later processing stage, i.e., when shapes are integrated and compared with internal prototypical object representations. The latter view is supported by studies with patients suffering from apperceptive agnosia due to right hemispheric lesions that included the parietal cortex. Such patients are impaired in recognizing objects presented atypically, while their object recognition in prototypical views is unimpaired, e.g., [10,11]. This suggests an involvement of the parietal cortex in view normalization in object recognition. In this context, view normalization refers to the process of transforming visual input so that it matches internally stored prototypes. An example of a process involved in transforming visual information is mental rotation. However, retinal representations are translated to prototypical views during view normalization to generate viewpoint invariant representations allowing for object recognition [12]. Conceptualized in this way, view normalization is also likely to comprise processes other than mental rotation. One of these potential mechanisms involves the scaling of incongruent object displays so that they match the prototypical, and thus congruent, object size. If view normalization indeed involves the transformation of incongruent size information, then one would expect activation in dorsal stream regions, more precisely in the right parietal cortex, when an object is presented at a retinal size that is inconsistent with its familiar object size.

To elucidate these processes, we investigated the neural underpinnings of where discrepant information regarding familiar and physical object size is resolved. First, functional MR imaging was performed on thirty-three healthy subjects presented with small and large (here: familiar) objects, which were displayed at small and large physical (here: display) sizes, hence generating congruent and incongruent display size/familiar size combinations.

The neural stage at which discrepancies between familiar and retinal object size are resolved indicates the processes underlying the familiar size incongruency effect. Activation in ventral visual areas would support the view that incongruent size information alters the encoding processes of prototypical object representations. Alternatively, an involvement of (right) parietal regions would suggest the involvement of size-based view normalization.

In the second experiment, the mismatch between familiar and display size was changed parametrically to account firstly for individual preferred viewing sizes, and secondly for the fact that within the two categories (small and large familiar size), there was substantial variation in terms of the real-world size.

## 2. Experiment 1

### 2.1. Materials and Methods

This experiment lasted 9 min and was embedded into a longer MRI session, which included a separate functional experiment and lasted about 45 min in total. Stimuli were presented on a 30 inch shielded LCD monitor (60 Hz) at a distance of 245 cm and seen via a mirror system installed on top of the head coil.

#### 2.1.1. Participants

A total of 33 healthy adults (12 women, mean age 26.8 years, age range: 18–44 years) participated in the current study. Three subjects had to be excluded, since the number of discarded trials (missed responses, errors, or reaction times faster than 200 ms) was more than two standard deviations larger than the group mean in the overall MRI session (remaining subjects: *n* = 30, 11 women, mean age 26.5 years, age range: 18–44 years). No neurological or psychiatric disorders were reported, and all subjects had normal or corrected-to-normal vision. Subjects were right-handed, as measured using the Edinburgh Handedness Inventory [13]. Following the Declaration of Helsinki, written informed consent was obtained before the experiment. Participants were remunerated for their time. The ethics committee of the German Society of Psychology (DGPS) approved the study.

#### 2.1.2. Task and Design

The experiment was performed to identify the brain regions involved in resolving discrepant information of familiar size and display size. Therefore, small and large familiar objects were shown at both small and large display sizes at ten different positions on the screen, resulting in a 2 × 2 factorial design (real-world size: small/large × display size: small/large). In order to investigate implicit size-processing and to separate task-related activation from perceptual processes, subjects were asked to indicate as quickly and as accurately as possible whether the object appeared on the left or the right side of the fixation cross by pressing a button with their corresponding index finger. Two hundred and forty trials were presented in four blocks separated by breaks of 5 s. An event-related design was used, with all trials shown in randomized order. Each stimulus was shown for 750 ms, followed by jittered inter-trial intervals, during which responses were recorded (ranging from 800 ms to 1700 ms, mean ITI = 1250 ms).

#### 2.1.3. Stimuli

**Figure 1:**
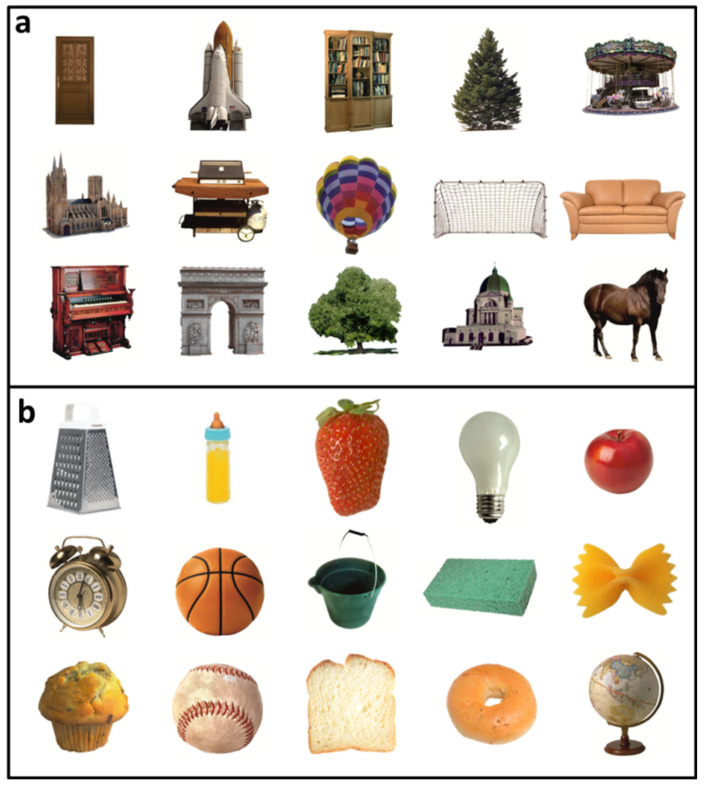
**Overview of the stimuli used in the current experiment.** Stimuli used in the current study were all made up of objects with either large (**a**) or small (**b**) real-world size as ranked by subjects in a study by [5].

**Figure 2:**
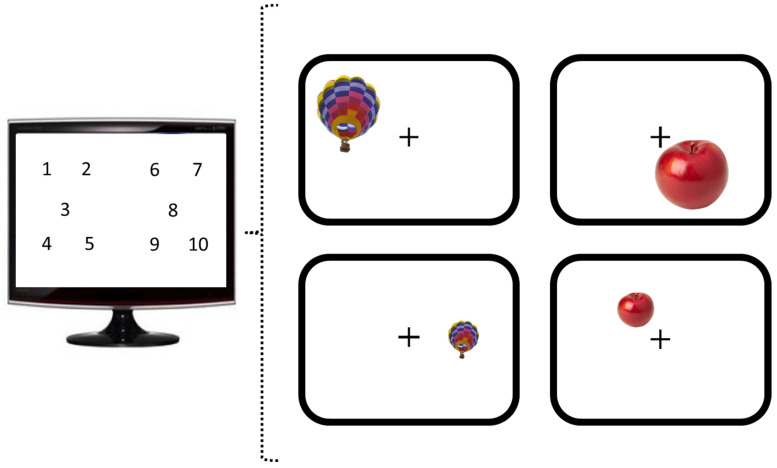
**Positions of stimulus appearance and illustration of the four experimental conditions.** Stimuli appeared on ten different positions on the screen. Images of single objects were shown at large (**top row**) and small (**bottom row**) display sizes.

Stimulus presentation was performed using the software Presentation (Version 17.0 Neurobehavioral Systems, Inc., Berkeley, CA, USA). Thirty objects were used (Figure 1), including fifteen objects that had been ranked to belong to the smallest 40% by subjects in the study mentioned above by [5] and fifteen objects that had been ranked among the largest 50%. Medium-sized objects were excluded for amplifying the difference in real-world size between the two object sets. Objects were presented either on the left (Figure 2, balanced across positions 1–5) or right side (Figure 2, balanced across positions 6–10) of the fixation cross. Each object was shown eight times: half of the time at a diagonal size of either 6.4° visual angle (display size large) and, during the other half of the trials, at a diagonal size of 3.2° visual angle (display size small), resulting in 240 trials. One hundred and sixty trials were selected for the analysis to ensure an equal number of trials for each of the eight combinations of position (left vs. right side of the screen) by familiar size (small vs. large) by display size (small vs. large).

#### 2.1.4. Data Analysis

Matlab (Version R2015a, The MathWorks, Inc., Natick, MA, USA) was employed to analyze the behavioral data. Trials without any response, with a wrong response, and trials with reaction times below 200 ms were excluded from the analyses.

### 2.2. MRI Data Acquisition and Analysis

#### 2.2.1. Scanning Parameters

A 3 Tesla TRIO MRI system (Siemens, Erlangen, Germany) was used to acquire functional imaging data. The images were collected using a T2* weighted echo-planar imaging (EPI) sequence with a repetition time (TR) of 2.2 s and an echo time (TE) of 30 ms. Two hundred and forty volumes, each including 36 axial slices, were measured with interleaved slice acquisition mode to obtain whole-brain coverage for every participant. Slice thickness was 3 mm with an interslice distance of 15% (i.e., 0.4 mm). The field of view was 200 mm, using a 64 × 64 image matrix resulting in a voxel size of 3.1 × 3.1 × 3.0 mm^3^. Additionally, we acquired structural images. A physician checked these for any abnormality.

#### 2.2.2. Preprocessing

Imaging data were preprocessed and analyzed using the Statistical Parametric Mapping software SPM (Version: SPM12, Wellcome Department of Imaging Neuroscience, London, UK) implemented in Matlab. The first nine images, acquired before the first stimuli appeared and before a steady BOLD signal was reached, were omitted from further analysis. All images were realigned to correct for subject movements during scanning and normalized to the segmented mean image. Finally, data were smoothed using a Gaussian kernel of 8 mm full-width half-maximum.

#### 2.2.3. Imaging Data Analysis

The blood oxygenation level-dependent (BOLD) response was modeled using a canonical hemodynamic response function and its time derivative. First, onset regressors representing the four experimental conditions were defined for each subject. These consisted of the time points where the object configurations appeared on the screen. As for the behavioral analysis, missing values, error trials, and trials with RTs faster than 200 ms were excluded (*mean* = 7.31 trials, *SD* = 5.31). The model included the onset regressors for the excluded trials and for the six head-movement parameters, plus the squared movement parameters as explanatory regressors. Initially, first-level contrasts for all conditions were specified by setting the regressor of interest to one and all other regressors to zero. Model estimation was performed for all voxels in the brain using the ANOVA flexible factorial design, as implemented in SPM12.

Next, second-level contrasts were calculated. First, the main effects were investigated for both display size ([display size large–small] and [display size small–large]) and familiar size ([familiar size large–small] and [familiar size small–large]). Please note, a combined main effect for familiar and display object size was not possible, since both main effects were orthogonalized (i.e., an object could have been small concerning familiar size and, at the same time, large in terms of display size). Therefore, a conjunction analysis was performed to identify activation changes caused by larger than smaller objects (and vice versa), irrespective of the type of size information. Finally, two interaction contrasts between the two factors, display and familiar size, were calculated to identify areas involved in processing congruency or incongruency in the display or familiar size. The first interaction contrast was to identify areas showing higher activation for objects displayed at their canonical size; that is, objects whose display and familiar size relative to the screen were congruent: [display small/familiar small + display large/familiar large] − [display small/familiar large + display large/familiar small]. The reverse contrast was to identify areas showing higher activation for incongruity between display and familiar size: [display small/familiar large + display large/familiar small] − [display small/familiar small + display large/familiar large]. A cluster-level correction was applied to those activations, which were significantly activated at *p* < 0.001 (uncorrected) using the extent voxel threshold of the smallest significant cluster at cluster level (FWE < 0.05).

### 2.3. Results

Behavioral data were analyzed to check whether subjects attended appropriately to the stimuli throughout the experiment. The subjects’ task was to indicate the location of the stimuli on the screen (left or right), and was hence unrelated to object size. This ensured that there was no confound by, e.g., explicit size judgments. The accuracy values for the location task were, on average, 96.96% (*SD* = 2.13%), indicating that subjects attended well.

Functional imaging results of the whole-brain second-level analysis showed no significant activations for smaller compared to larger objects, neither at the level of familiar size nor the level of physical size, nor looking at the conjunction of both. However, the opposite main effects showed large activation clusters within the early visual cortex and ventral visual stream.

**Figure 3:**
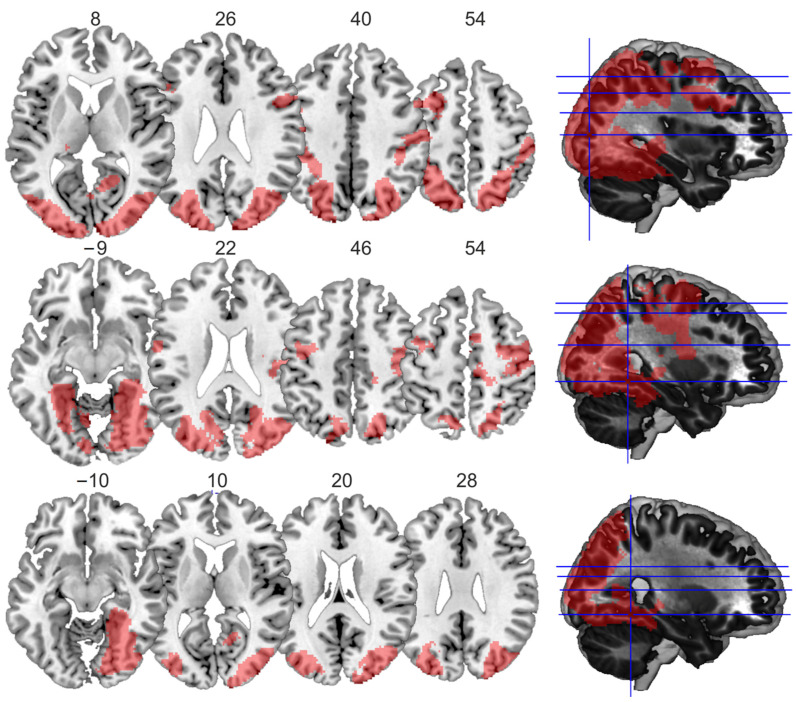
**Visualization of the main effects and the conjunction.** Main effects for display size (**top row**), familiar size (**middle row**), and the conjunction contrast (**bottom row**), as visualized using MRIcron. Slices were chosen based on the peak coordinates of the significant clusters, and the numbers correspond to the z-coordinates of the chosen slices, which are also illustrated in the respective rendered brains in the right column.

First, the main effect for large display size yielded four large clusters, one spreading from both the left and right occipital gyrus ventrally along the fusiform gyrus and dorsally up into the parietal cortex, and three smaller ones within the frontal cortex (Table 1a, Figure 3). The main effect for large familiar size showed similar activations within two clusters, one within the left and one within the right hemisphere, spreading from the occipital cortex to the fusiform gyrus, and three smaller clusters around the left and right central sulcus and in the medial frontal cortex (Table 1b, Figure 3). The conjunction analysis of both main effects identified two large clusters, one in the left and one in the right hemisphere. Both involved the fusiform gyrus and middle occipital cortex (Table 1c, Figure 3).

**Figure 4:**
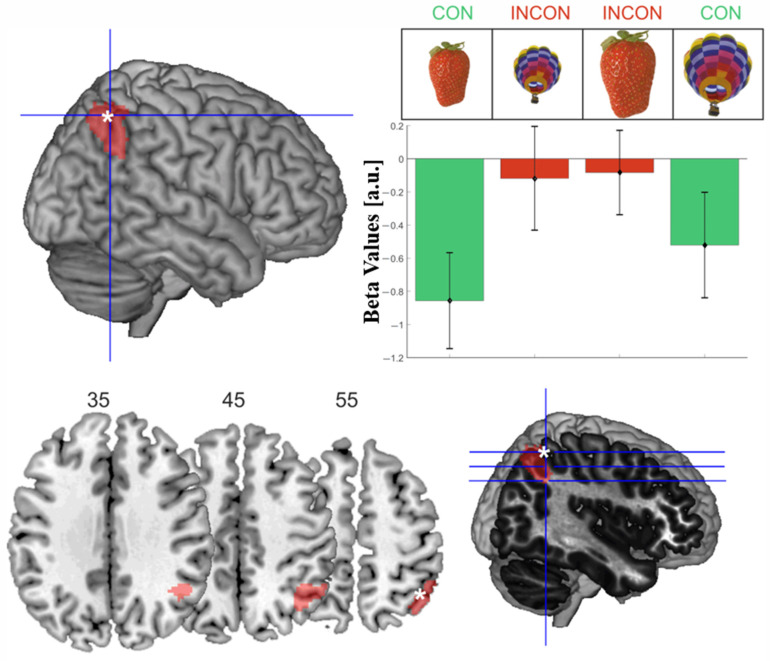
**Visualization of the activations for the interaction contrast**. More activation for incongruent > congruent objects was found within the right intraparietal sulcus. (**Top left**) white asterisk indicates the activation’s peak voxel (44, −56, 56); (**top right**) beta values extracted from the peak voxel are plotted for the four conditions; (**bottom row**) three exemplary slices were visualized using MRIcron, corresponding to the blue lines on the right with the peak voxel indicated by the white asterisk.

The interaction contrast identified one cluster peaking in the right intraparietal sulcus (IPS) extending into the inferior parietal lobule and the angular gyrus, which showed significantly stronger activation for incongruent compared to congruent object information configurations (Table 1d, Figure 4). Beta values were negative in all four conditions due to relatively short baseline epochs for brain signals to return to the baseline. The extracted beta values for the peak voxel revealed that within the right IPS, incongruent objects showed relatively stronger activation than congruent object information.

### 2.4. Discussion Experiment 1

Enhanced activation was found for the large display size compared to the small display size in the occipital cortex, extending ventrally to the fusiform gyrus and dorsally to the parietal cortex. Large display-size objects occupy large retinal areas, hence inducing larger activation in the retinotopically organized visual areas. Moreover, large familiar sizes also showed similar activation patterns. Therefore, we replicated earlier findings showing that the ventral stream stores object knowledge, and thus holds prototypical object representations [15]: Larger compared to smaller objects activated early visual and ventral visual areas irrespective of whether object size was defined in terms of display or familiar size.

These results are also consistent with previous findings by Konkle and Oliva [9], who reported a medial to lateral organization for large and small real-world objects within ventral visual areas and identified areas within the parahippocampal gyrus, which showed a preference for large real-world objects and an overall higher activation for objects displayed in a larger size. However, as opposed to the study by Konkle and Oliva [9], there were no circumscribed regions with higher activation for large versus small objects, and the visual activation was more widespread. Most likely, this can be ascribed to the fact that objects in the current study appeared in different positions on the screen, leading to position uncertainty and a larger overall eccentricity of visual activation.

Importantly, based on the results of the whole-brain analysis and the higher IPS activation for incongruent as opposed to congruent objects, we hypothesized that the right IPS processes differences between individual prototypical size information and the actual display size of an object. Taking into account the previously introduced patient studies, which found the right parietal cortex was involved in matching atypically presented visual objects with prototypical object representations [10,11,16], right IPS also constitutes a candidate region for a size view-normalization process. However, given that within the categories of small and large, familiar size objects varied still substantially in terms of their real-world size, a follow-up study was performed to investigate potential parametric changes within the right IPS, supporting the idea of view normalization.

## 3. Experiment 2

Experiment 2 was performed to test further the right IPS’s involvement in processing differences between individual prototypical size information and the actual display size of an object, as suggested by the data from Experiment 1. A parametric fMRI design was adopted which probes the view-normalization process explicitly, without comparing across real-world sizes as in Experiment 1. It also accounted first for individual preferred viewing sizes, and secondly for the fact that within the two categories (small and large familiar size) there was substantial variation about the real-world size. A region-of-interest approach was employed to perform the analyses, since we had a clear a priori hypothesis based on Experiment 1.

### 3.1. Materials and Methods

The fMRI session lasted 9 min, and the setup was the same as for the first part of the study. Again, stimuli were presented on a 30 inch shielded LCD monitor (60 Hz) at a distance of 245 cm and seen via a mirror system installed on top of the head coil.

#### 3.1.1. Participants

A total of 15 healthy adults (10 women, mean age 30.93 years, age range: 22–45 years) participated in Experiment 2. Two subjects had to be excluded due to too many missing responses, hence reducing the number of trials below the minimum number of trials required for a robust analysis (more than two standard deviations less than the group mean; remaining subjects: n = 13, 10 women, mean age 31.08 years, age range: 22–45 years). No neurological or psychiatric disorders were reported, and all subjects had normal or corrected-to-normal vision. As for the first experiment, subjects were right-handed, as measured using the Edinburgh Handedness Inventory [13]. Written informed consent was obtained before the experiment, which followed the Declaration of Helsinki. Participants were remunerated for their time. The ethics committee of the German Society of Psychology (DGPS) approved the study.

#### 3.1.2. Design

Task, stimuli, timing, and presentation duration were identical to the first experiment. The same 30 objects (Figure 1) appeared at the same ten different positions on the screen (Figure 2). Moreover, the same inclusion criteria for subjects and single trials were applied as in Experiment 1 (number of included trials of the final sample: *mean* = 235/240, *SD* = 4.27). However, the design differed insofar that the congruency of canonical size and the display size now varied parametrically. Each object was presented at four display sizes (instead of two in Experiment 1), once on the left and right side of the screen. The three factors position (1–10), object (1–30), and display size (1–4) were balanced carefully.

Moreover, before the functional measurement, individual canonical sizes for each subject were derived in a procedure similar to Experiment 3 reported in Konkle and Oliva [5]: already lying in the scanner, subjects saw all 30 objects in an intermingled order, with each object appearing twice. Using the response buttons underneath their left and right index finger, subjects could decrease and increase the display size until objects “looked best” to them (a procedure similar to [5,17]). The mean display size of both appearances was stored as the canonical size of that particular object for the individual subject.

#### 3.1.3. Data Analysis

To analyze the behavioral data, the consistency of size preference ratings was analyzed first. This was achieved by looking at the difference in size rating for each subject at the two presentation times. Next, we investigated whether the subject’s individual size preference ratings were related to the logarithm of the actual object size, as shown previously [5]. Therefore, a regression analysis was performed using the free statistical software R (Version 3.1.3, R Foundation for Statistical Computing, Vienna, Austria). The regression analysis aimed at testing whether logarithm of its true size predicted the individual canonical size of any particular object.

### 3.2. MRI Data Acquisition and Analysis

Scanner and scanning parameters were identical to the first experiment. All structural images were again checked by a physician, and no abnormalities were found. If not otherwise specified, the analysis steps were as described above. The model estimated on the first level differed slightly insofar that, apart from the same regressors of no interest that were also used in the first experiment, there were only two regressors of interest, one including the onsets of all trials and one including the parametric modulation. Parametric modulation on each trial was calculated as the difference between the individual canonical size (derived from the size preference rating before Experiment 2) and the current display size. For the second-level analysis, a one-sample t-test was performed to investigate activation changes associated with the parametric changes, i.e., the difference between the preferred and the actual display size of each object (*size mismatch*). Since Experiment 2 was performed to test a hypothesis based on Experiment 1, a region-of-interest approach was employed. The cluster-level corrected (*p* < 0.001) activation in the right IPS, which was found for the interaction contrast in Experiment 1 (Table 1d, Figure 4), was used as a mask during the second-level analysis of Experiment 2.

### 3.3. Results

Analyzing the behavioral data showed considerable internal consistency in the size preference ratings. Ratings differed on average 12.41% (*SD* = 8.12%) between the two judgments performed for each of the thirty objects. Moreover, the accuracy of the localization task which was performed to maintain subjects’ attention was as high as 98.17% (*SD* = 1.78%) with 0.71% errors (*SD* = 0.52%) and 1.12% misses (*SD* = 1.51%), indicating that subjects attended well to all stimuli.

Next, the results of the regression analysis as calculated in R showed that individual canonical size of any particular object was predicted by the logarithm of its true size: The regression was significant, looking at each subject separately (all F (1,28) > 11.01; all *p* < 0.003). A one-sample t-test showed that the slope of all regression lines was significantly larger than zero (mean slope = 0.19, t(12) = 8.72, *p* < 0.001). This indicates that subjects’ preferred sizes were related to real-world sizes and internally stored prototypes. These results align with previous studies [5] and show that subjects do have a viewing preference that corresponds to internally stored canonical sizes for real-world objects.

After validating the behavioral results—that is, the individual preferred sizes—functional data analysis was performed. The region-of-interest analysis of the right IPS cluster (as defined based on Experiment 1) showed that the parametric modulation by size mismatch was associated with higher activation in two clusters (Table 2).

### 3.4. Discussion Experiment 2

Experiment 2 supports the hypothesis that the right IPS contributes to resolving discrepancies between different types of size information. In this case, the discrepancies related to quantitative differences between the actual viewing size (here: display size) and the individual canonical size of an object. The latter is defined as the preferred viewing size of a familiar object and is closely linked to its proper size, as shown by the significant relationship with the logarithm of the proper size found in the current and previous studies [5]. The fact that parametric changes of the degree of mismatch between the two measures were associated with increased activation of right IPS, as identified in Experiment 1, supports the idea that viewing size is normalized within this area to match internally stored prototypes.

## 4. General Discussion

The current study examined the neural mechanisms of integrating display size and familiar size in healthy human subjects. To this end, we investigated whether familiar objects presented at incongruent display sizes (i.e., large familiar objects shown at a small size on the screen) would recruit parietal regions associated with spatial processing or ventral visual regions associated with object knowledge.

Furthermore, the interaction contrast identified the brain regions whose activation pattern reflected interference between both types of size information (i.e., display size and familiar size). If this interference occurred within ventral visual brain regions, known to represent display size and familiar size, this could be taken as evidence that interference is resolved at early representational levels. In other words, an involvement of ventral visual regions in the processing of discrepancies between both types of size information would suggest those discrepancies were resolved when retrieving object information from the ventral visual cortex, leaving object recognition processes unaffected. Alternatively, as introduced before, activation in brain regions known to be involved in object agnosia, such as the parietal cortex, would speak in favor of the involvement of object recognition processes, e.g., matching active representations with stored internal prototypical representations.

Our results provide evidence for the second hypothesis. In Experiment 1, objects presented at an incongruent familiar size and display size were associated with higher neural activity within the right parietal cortex, more precisely the right IPS, suggesting that the right parietal cortex is critically involved in resolving mismatches arising from inconsistencies between incoming perceptual information and internally stored object representations. This right parietal activation may represent several cognitive processes. Although it has been argued that size incongruencies can cause a Stroop-like interference, the current activations are different from those commonly associated with cognitive control and Stroop tasks. These have been associated with both prefrontal and left [18,19,20,21,22] or bilateral parietal regions [23,24]. In contrast, the parietal activation reported in the current study was found in the right hemisphere only.

It has been suggested that the right parietal cortex plays a role in processes serving object recognition. For instance, the GABA level of the parietal cortex selectively correlated with the size illusion magnitude, suggesting the parietal cortex has a role in size perception [25]. Further, right parietal cortex lesions have also been associated with deficits in the perception of mirrored and rotated stimuli [26]. Consistent with these findings, patients suffering from apperceptive agnosia due to right parietal damage show deficient object recognition when objects are viewed from atypical viewpoints [10,11,16]. Taken together, these findings suggest a critical role of the right parietal cortex in transforming incoming object-related spatial information to match internally stored prototypical information. This view is supported by electrophysiological data from macaques that demonstrate that IPS integrates sensorimotor and visual information to guide and control (object-related) action in space, (for a review, see [27]). Based on patient data, Riddoch and Humphreys [12] hypothesized that object recognition involves a “view normalization process” that transforms retinal representations to prototypical views, thereby generating viewpoint invariant representations allowing for object recognition. The current data suggest that these prototypical object representations are not restricted to prototypical viewpoints, but include other prototypical features, such as typical viewing distance and typical viewing size. It has been found that IPS is involved in associating different images of famous faces as belonging to the same identity, which makes IPS a candidate for matching incoming information to internally stored prototypes, going beyond the process of mere mental rotation [28]. Moreover, the right parietal lobe is specialized in spatial processing, i.e., perceiving object orientation and translating objects in space [26,29], and size and distance perception are closely linked concepts highly relevant for navigating in space. Therefore, view normalization is most likely not limited to viewpoint normalization, but also includes the normalization of typical viewing size and typical viewing distance. In the current study, both concepts are closely linked.

It is conceivable that the activations observed reflect differences in terms of *typical* viewing distance, given that large familiar objects (e.g., a hot air balloon) are typically seen further away than small familiar objects (e.g., a cup), which implies that incongruent objects are closer to their typical viewing distance than congruent objects. Accordingly, view normalization may relate to viewing distance and object size information. In any case, we can assume that both require a normalization process before they match the internally stored object representations in the ventral visual cortex. More specifically, the incongruent objects require more normalization than the congruent objects to match stored prototypical representations. This requirement is compatible with the observed higher right IPS activation for incongruent as compared to congruent objects. However, Experiment 1 did not quantitatively vary the degree of incongruency, i.e., the degree of mismatch between familiar and retinal size. Apart from that, individually stored prototypical sizes may differ between subjects, leading to more or fewer (in)congruent trials. In order to address both issues, Experiment 2 was performed, addressing the assumption of view normalization in the right IPS by parametrically varying the size mismatch and testing whether a greater size mismatch was associated with more activation within this region. The region-of-interest analysis showed that an increased mismatch between an object’s actual display size and an individual preferred viewing size of that same object was associated with increased activation within the predefined region in the right IPS.

In sum, the present data suggest that the right parietal cortex performs view normalization of object representations not only for atypical viewpoints, as shown in patient studies, but also for atypical sizes or distances. We conclude that disruption of the right IPS in the healthy brain should cause impairment in view normalization for objects shown at incongruent sizes. IPS has been previously associated with the processing of magnitudes such as numerosity, object size, and illusionary size changes [30,31,32,33,34].

The view that dorsal brain regions are involved in accounting for retinal and familiar size information in the context of object recognition is consistent with findings from a recent study that investigated an interesting phenomenon known as the “real-object advantage” (ROA) in visual agnosia patients [35]. In this study, patients suffering from visual agnosia due to ventral stream damage showed preserved object recognition when the physical size of the objects matched their real-world sizes. The performance dropped severely when objects were presented in either too large or too small sizes. The authors suggested that the dorsal visual cortex mediates object size information and that this information, together with a detailed representation of object shape, also subserved by the dorsal cortex, serves as the basis of the ROA. Hence, size normalization may require interaction between ventral and dorsal stream regions, and lesions in either of these regions may prevent effective size normalization.

Future studies using repetitive transcranial magnetic stimulation (rTMS) or transcranial direct current stimulation (tDCS) could investigate whether transient dysfunction of right IPS equally interferes with tasks requiring view normalization for object recognition, such as those used in the previously introduced patient studies [11,16]. Based on our findings, such transient lesions are expected to introduce impaired object recognition for objects displayed at incongruent display and familiar sizes.

## Figures and Tables

**Table 1:** **Results of the second-level analysis of Experiment 1.** Coordinates were defined within MNI space, and only the peak coordinates for each respective cluster are reported. Activations were all significant at *p* < 0.001 (uncorrected), with an extent voxel threshold that allows including the number of voxels of the smallest significant cluster at *p* < 0.05 (FWE). Regions were identified using the anatomy toolbox, as implemented in SPM 12 [14].

Contrast	Regions	Cluster Size	Side	x	y	z	Z-Score
(a) Main effect: Display size large > small	Middle occipital gyrus Inferior occipital gyrus Inferior temporal gyrus	26,012	R (L	34 −32	−84 −90	8 12) *	Inf
	Precentral gyrus Inferior frontal gyrus Middle frontal gyrus	832	L	−54	4	40	5.50
	Middle frontal gyrus Superior frontal gyrus	638	L	−28	10	54	4.64
	Inferior frontal gyrus Precentral gyrus	268	R	52	12	26	4.39
(b) Main effect: Familiar size large > small	Fusiform gyrus Parahippocampal gyrus Middle occipital gyrus	8271	R	30	−50	−8	Inf
	Parahippocampal gyrus Fusiform gyrus Middle occipital gyrus Calcarine gyrus	4919	L	−28	−42	−10	Inf
	Rolandic operculum Putamen Precentral gyrus Middle frontal gyrus Insula Superior frontal gyrus Postcentral gyrus	2548	R	48	−18	22	5.13
	Precentral gyrus Postcentral gyrus Middle frontal gyrus	889	L	−40	−8	46	4.36
	Posterior-medial frontal	497	R	14	−26	54	4.20
(c) Conjunction of (a) and (b)	Fusiform gyrus Middle occipital gyrus	6536	R	28	−50	−10	Inf
	Middle occipital gyrus Precuneus Superior parietal lobe Cuneus	1980	L	−34	−82	28	6.16
(d) Interaction: Incongruent display and familiar size > congruent display and familiar size	Intraparietal Sulcus (IPS)	633	R	44	−56	56	4.38

* Note: Since this cluster spread bilaterally in a symmetric manner, the highest voxel within the left hemisphere is reported here in parentheses.

**Table 2:** **Results of the second-level analysis of Experiment 2.** Coordinates were defined within MNI space, and activations were all significant at *p* < 0.05 (uncorrected) with a one tailed test using a region-of-interest-based approach with an inclusive mask derived from Experiment 1 (cluster in Table 1d).

Contrast	Regions	Cluster Size	Side	x	y	z	Z-Score
Parametric modulation by *size mismatch*	Intraparietal Sulcus (IPS)	26	R	46	−56	32	2.13
		4	R	54	−56	42	1.92

## Data Availability

Data are available from the corresponding author upon reasonable request, for research purposes only.
